# Health Risks Associated With Excessive Exposure to Solar Ultraviolet Radiation Among Outdoor Workers in South Africa: An Overview

**DOI:** 10.3389/fpubh.2021.678680

**Published:** 2021-04-28

**Authors:** Caradee Y. Wright, Mary Norval

**Affiliations:** ^1^Environment and Health Research Unit, South African Medical Research Council, Pretoria, South Africa; ^2^Department of Geography, Geoinformatics and Meteorology, University of Pretoria, Pretoria, South Africa; ^3^Biomedical Sciences, University of Edinburgh Medical School, Edinburgh, United Kingdom

**Keywords:** cataract, employment, environmental health, skin cancer, sun exposure, keratinocyte cancers, melanoma, personal sun safety

## Abstract

Exposure of outdoor workers to high levels of solar ultraviolet radiation (UVR) poses significant, well-known health risks including skin cancer and eye diseases. In South Africa, little is known about how many workers are potentially overexposed to solar UVR and what the associated impacts on their health might be. In this overview, the geography and solar UVR environment in South Africa are considered, as well as the different outdoor occupational groups likely to be affected by excessive solar UVR exposure. Sunburn, pterygium, cataract, keratinocyte cancers, and melanoma are discussed in the context of outdoor workers. Few studies in South Africa have considered these health issues and the most effective ways to reduce solar UVR exposure for those working outside. Several countries have developed policies and guidelines to support sun safety in the workplace which include training and education, in addition to the provision of personal protective equipment and managerial support. Several gaps in occupational sun protection and workplace sun safety for South Africa are identified. Legislation needs to recognize solar UVR exposure as an occupational health hazard, with sun safety guidelines and training provided for employers and employees.

## Introduction to South Africa

Outdoor workers are particularly vulnerable to acute and chronic health risks from excess exposure to solar ultraviolet radiation (UVR) (1). The skin and eyes are the most common target organs. It is of considerable interest to assess the risk for outdoor workers in South Africa as this country is subtropical, has a multi-ethnic population and the UV Index can reach 13 in the summer months (a UV Index of 11+ is considered extreme) (2). In this overview, we undertook a systematic search initially mainly using PubMed with the terms “South Africa,” “outdoor workers,” “solar UV radiation”/“sun exposure,” “skin diseases”/“eye diseases” and then each category of outdoor worker and each category of disease separately. References listed in related papers were also retrieved. We present the geography of South Africa and its climate, together with a summary of the population and outdoor worker groups. An account is then given of the ocular and cutaneous health risks associated with excess sun exposure of outdoor workers in South Africa, followed by studies examining sun protection. The final section considers actions needed to prevent the adverse health risks from excess sun exposure in the country.

## Geography and Climate

South Africa is situated in the midlatitudes between 22°S and 35°S. Its topography comprises coastal plains and a large, central plateau, the Highveld, located in the interior of the country at about 1,200 m. Frequent high pressure over the plateau leads to relatively cloudless skies throughout the year which, together with the high altitude, contributes to high ambient solar UVR levels. Table 1 shows the maximum, minimum and mean UV Index, sunshine hours and ambient temperature in summer and winter in Cape Town, Durban and Pretoria.

**Table 1:** Maximum, minimum and mean UV Index, sunshine hours and ambient temperature in summer and winter in Cape Town, Durban, and Pretoria (weather-and-climate.com; weather-atlas.com).

	**Cape Town**	**Durban**	**Pretoria**
Latitude	33.9°S	29.9°S	25.7°S
Altitude (m)	0–300	8	1,339
**Summer temperature (°C)**			
Maximum	26	28	29
Minimum	16	21	18
Mean	23	25	25
**Winter temperature (°C)**			
Maximum	18	23	19
Minimum	7	12	5
Mean	13	18	13
**Summer sunshine hours**			
Mean daily	10	6	8
**Winter sunshine hours**			
Mean daily	6	8	10
**UV Index**			
Summer	9–10	12	11+
Winter	2–3	4–5	4–6

## Population Groups and Outdoor Occupations

**Figure 1:**
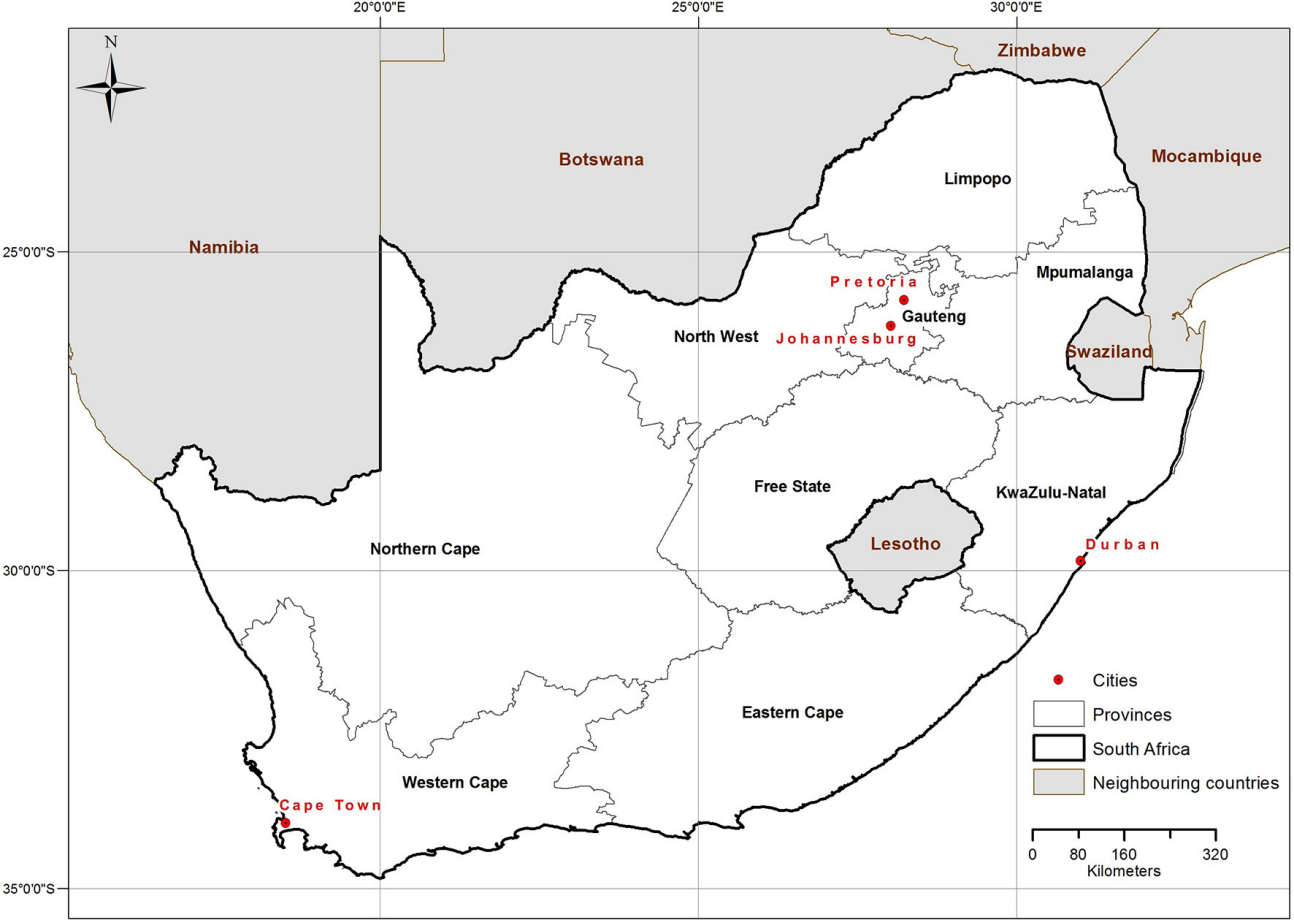
Map of South Africa indicating the nine provinces and the four major cities.

Four groups formally delineate the population of South Africa, namely Black African, White, Indian/Asian and Coloured [mixed European (White) and Black African or Asian ancestry]. Of the 59.6 million population in 2020, 80% were Black African, 8% White, 3% Indian/Asian and 9% Coloured (3). The country is divided into nine provinces (Figure 1) with about 40% of the population residing in the four coastal provinces, Northern Cape, Western Cape, Eastern Cape and KwaZulu-Natal, and the largest percentage of the population (26%) living in the inland province of Gauteng.

About 14 million people of working age (18–65 years) are employed in all sectors in South Africa (4). Of these, approximately 4 million people work outdoors either in formal or informal jobs. About 250 000 people are employed in forestry, 150,000 in fishing, 885,000 in formal agriculture and 3 million in subsistence or household agriculture (5–8). It is not possible to determine exact numbers of people working outdoors in mining and construction since they may be underground and indoors, respectively for all or part of their employment. However, it is assumed that a considerable proportion of South African construction workers stay outdoors for at least part of their working day, as has been shown in a study based in Denmark (9). In addition, those working in surface, open-pit mines are likely to experience significant sun exposure. Out of approximately 92,000 employed as coal miners in South Africa in 2019, half worked in open-pit mines, and about one-third of the 95,000 working in gold mines (10).

Solar UVR exposure of outdoor workers has been measured for different occupations around the world with few studies carried out in South Africa. Construction workers in Australia were exposed to a daily dose of 10 standard erythemal doses (SEDs where 1 SED = 100 Jm^−2^) (11). Farmers in Italy received on average 15 SED per day or about 80% of the ambient solar UVR (12). Similarly, a South African school groundsman/gardener was exposed to 80% (4 SED) of the ambient solar UVR per day (13) while farmworkers were exposed to 46% (8–12 SED) of the ambient solar UVR per day (14).

The factors influencing how much solar UVR an outdoor worker receives are environmental, including latitude, altitude, cloud cover, solar zenith angle, stratospheric ozone and albedo, and occupational relating to the type of work activity, length of time spent outside, and provision of physical sun protection infrastructure, such as shade (1). Individual factors include personal attitudes and sun protection used. In addition, skin phototype is an important parameter: those people with fair skin burn easily in response to solar UVR exposure and do not tan, while the presence of melanin in those with deeply pigmented skin offers protection against sunburn and other detrimental health aspects of solar UVR exposure (15, 16).

## Health Risks Associated With Excessive Solar UVR Exposure Among Outdoor Workers in South Africa

Several eye and skin diseases globally are associated with sunlight exposure. Some are classed as acute, becoming evident several hours after a high dose of solar irradiation. Details are provided below of acute conditions in both the eye and skin. Others occur as a result of chronic exposure to solar radiation. The major chronic sun-associated diseases in the eye are non-melanoma skin cancer (NMSC) of the lid and conjunctiva, ocular melanoma, cataract, pterygium, climatic droplet keratopathy (epithelial degeneration) and pinguecula (local degeneration of conjunctiva). The major chronic sun-associated diseases in the skin are NMSC and cutaneous melanoma (CM). Below, details are provided of pterygium, cataract, NMSC, and CM. It should be noted that epidemiological studies of these diseases in South Africa are rare but, considering the frequent high UV Index in this country, in association with warm temperatures, clear skies and reflective terrain or water, it is likely that solar UVR-induced ocular and cutaneous damage occurs, which may present particular health risks in those who work outdoors.

## Acute Effects of Solar UVR on The Eye and Skin

Sunburn is well-recognised following excessive sun exposure, with photoconjunctivitis (inflammation of the conjunctiva) and photokeratitis (inflammation of the cornea) on the surface of the eye also considered as sunburn. Individuals with fair skin are more susceptible to sunburn than those with pigmented skin; (16) indeed, it has been calculated that there is an approximate 10-fold increase in the erythemal sensitivity of white skin compared with black skin (17) but those in the latter category can still get sunburnt.

Rosenthal et al. estimated that an outdoor worker was likely to receive 10–70% of the total ambient daily solar UVR, depending on the time spent in the sun that day (18). A figure of 20% of the total daily ambient solar UVR, as measured by ground-based instruments, was converted into possible exposures of outdoor workers by skin type and season at two locations in South Africa, Durban (latitude 30°S) and Cape Point (34°S) (19). It was concluded that there was a risk of sunburn for outdoors workers in both sites throughout the year for all the ethnic South African populations, except in mid-winter for those with deeply pigmented skin. Finally, a pilot study, prompted by an increase in ambient temperatures associated with global warming, reported that those working outdoors in sun-exposed conditions in hot parts of South Africa experienced painful eyes and blurring of vision which may indicate ocular sunburn (20).

These reports, while few in number, do show the real possibility of outdoor workers in South Africa getting sunburnt on their eyes or exposed skin during the course of their work. This is of concern, not only for the immediate health of the individual but because such episodes, when repeated, are likely to increase the risk of skin cancer in later life (see sections below).

## Chronic Effects of Solar UVR on The Eye

### Pterygium

Pterygium is a wing-shaped invasive growth on the conjunctiva that frequently starts at the corner near the nose, causing the eye to feel itchy and burning. It can lead to extreme discomfort as it progresses and to blurred vision if it covers the pupil. Pterygium is one of the commonest eye disorders, with a mean age of development of 44 years. The population attributed factor of pterygium due to solar UVR exposure was calculated as 42–74% in 2006 and, as outdoor work is one of the relevant risk factors, the suggestion was made that pterygium should be considered as an occupational disease (21). The most recent meta-analysis regarding pterygium included 68 articles from 24 countries, although none from South Africa (22). Prevalence was estimated as 12% in the total population globally and slightly higher in men than in women. Exposure to sunlight was the major environmental risk factor with odds ratios of 1.24 for sunlight exposure longer than 5 h daily, 1.45 living in rural areas, 1.46 outdoor occupations and 0.47 if sunglasses were worn.

Few studies have been published which provide detailed information about pteryium in South Africa. Corneal diseases, mainly pterygium and climatic droplet keratopathy, were present in 20% of Coloured patients in a local community in northwest Limpopo (23). Angurin et al. proposed that exposure to sunlight could be a trigger for pterygium development in genetically predisposed Black Africans living in rural Limpopo province (24). In Ibadan, Nigeria, the prevalence of pterygium in patients attending an eye clinic was 9% with 65% of those being outdoor workers (25).

### Cataract

There are three types of age-related cataract based on the location of the lens opacities: nuclear which is the most frequent, followed by cortical and then posterior subcapsular cataract, the least frequent. Epidemiological studies many years ago linked sunlight exposure and cataract development (26, 27). Subsequently exposure to solar UVR was recognised by the World Health Organization (WHO) as the major environmental risk factor for cortical cataract (21). A systematic review in 2018 discovered that 15 studies had been published between 1997 and 2017 in which the risk of cataract was evaluated in the context of outdoor work (28). Twelve of these showed a positive association between long-term occupational solar UVR and cortical cataract with some evidence for nuclear cataract too. A meta-analysis to enable a relative risk to be calculated was not possible as the design of each study was different, and the methods used to estimate occupational UVR exposure were not exact. Therefore, although no study has been carried out that monitors the risk of cataract development in outdoor workers in South Africa, there is sufficient evidence from many other countries to indicate that this is highly likely.

Cataract accounts for about 50% of cases of blindness globally and sub-Saharan Africa has the highest regional burden of blindness at 20% of the world's cases and only 11% of the world's population. The prevalence of self-reported cataract in South Africa was 4.4% from data collected in 2007–2008 (29). Early studies in South Africa showed an annual incidence of cataract blindness of 0.14% with a prevalence of 0.6% in a rural population in KwaZulu-Natal, (30) and cataracts were the cause of loss of vision in 60% of blind Black Africans in rural Northern Transvaal (31). In the only recent study on cataract based in South Africa, Khoza et al. estimated that the prevalence of cataract was 67.4% in those aged over 18 years living in rural villages in Vhembe district, Northern Limpopo (32). It is known that cataract formation begins earlier in African populations than in comparable populations in India and USA, (33) and that it is more common in rural than urban areas (34, 35).

## Chronic Effects of Solar UVR on Skin

### Non-melanoma Skin Cancers (NMSCs)

These comprise squamous cell carcinomas (SCCs) and basal cell carcinomas (BCCs), also called the keratinocyte cancers. Actinic (or solar) keratosis, which presents as a red scaly patch on sun exposed body sites, is considered an early *in situ* form of SCC. Both BCCs and SCCs are disfiguring and debilitating, with SCCs occasionally becoming invasive and life-threatening if left untreated. NMSCs have the highest incidence of any cancer in Caucasian populations (35). They occur in people of all skin colours but particularly in those with fair skin as the high content of cutaneous eumelanin in pigmented skin provides substantial protection, estimated as 13-fold in African Americans compared with the White American population (36).

Exposure to solar UVR is the major environmental risk factor for both BCC and SCC (37). Intermittent high solar UVR exposures, especially in childhood and adolescence, together with chronic exposure, promote the development of BCCs; cumulative life-time exposure promotes the development of SCCs (38). In the context of outdoor workers, it should be noted that using data obtained from personal UV exposure may provide a more valid association with the risk of skin cancer development than relying on occupation title as a proxy.

In South Africa in 2000–2004, the age-standardised annual incidence of BCC per 100,000 was 3.0 and 1.7 in Black African men and women respectively, and 198 and 113 in White men and women respectively, while the incidence of SCC was 3.0 and 1.6 in Black African men and women respectively, and 70 and 32 in White men and women respectively (39). BCCs in people of all skin colours occur predominantly on sun-exposed body areas and on the back. SCCs in Black Africans develop mainly on the lower limbs but in Whites are found on body sites most exposed to the sun, such as the face and backs of the hands. As the diagnosis of NMSCs in South Africa is made solely on the basis of histological findings, under-reporting is certain as local treatment of lesions is often undertaken without first collecting biopsies or individuals do not recognise their own skin tumours. Thus, it is difficult to detect trends in incidence although the number of cases per year globally has increased markedly in recent years and South Africa has probably followed this trend, at least in the White population group.

Regarding the effect of solar UVR on the risk of NMSC in outdoor workers, no reports based in South Africa have been published. However, there is compelling evidence from a systematic review and meta-analysis that included 18 studies based in various locations in Europe, North America and Australia (40). There was an increased risk of SCC in those with occupational solar UVR exposure compared with those not having occupational solar UVR exposure: the odds ratio was 1.77. Furthermore, the strength of the association increased with decreasing latitude and thus higher ambient solar UVR. In a similar fashion, the relationship between BCC in outdoor workers and solar UVR was analysed in another systematic review and meta-analysis (41). There was a 40% increased risk of BCC in outdoor workers compared with indoor workers or the general public, and a strong inverse relationship between occupational solar UVR exposure and BCC risk with latitude. Recent studies have indicated that actinic keratoses are twice as common in those who worked outdoors in Denmark compared with indoor workers, (9) and that outdoor workers in Italy had a significantly higher incidence of NMSC or actinic keratosis than those with no outdoor work (42).

Despite the lack of direct evidence from South Africa and especially when the frequent high UV Index allied with hot temperatures may make wearing sun protective clothing less likely, it would be astonishing if there was not a considerable risk of outdoor workers developing NMSC and actinic keratosis, although Black Africans will be at lower risk than their White counterparts due to their pigmented skin.

### Cutaneous Melanoma (CM)

CMs are the least common of the skin cancers in people with fair skin but generally outnumber BCCs in those with pigmented skin. CMs account for more than 80% of deaths from skin tumours with late presentation and a more aggressive course in pigmented compared with fair skin (36). CMs occur most frequently on the backs of men, the legs of women and sun exposed body sites in the elderly in those with fair skin, (43) while they present mainly as acral lentigenous lesions on the palms of the hands, soles of the feet and around nails in those with pigmented skin (44). A survey in 2020 covering 31 countries, although none in Africa, found a general increase in the incidence of CM and mortality since the 1960s, especially in men, with an indication that these rates may be stabilising in the past decade in younger birth cohorts (45).

Data from the National Cancer Registry of South Africa showed that the age-standardised incidence per year of CM between 2005 and 2015 per 100,000 people was 0.5 in the Black African population and 23.2 in the White population, (46) thus demonstrating the protection offered by eumelanin in pigmented skin as one factor explaining the substantial difference in incidence (47). Over 800 deaths from CM were registered in South Africa in 2016 (48). Although the lack of a comprehensive population-based death registry in South Africa limits an accurate assessment of trends in CM mortality, an increase of about 3% in the White population between 1999 and 2014 was estimated, with no change in the Black African population (49).

Exposure to solar UVR as a risk factor for CM is complex. In people with fair skin, a dual pathway has been proposed whereby naevi, initiated by early sun exposure and promoted by intermittent high sun exposure thereafter, represents one route, and chronic sun exposure in sun-sensitive individuals represents a second route (50). As the majority of CMs in Black Africans develop on sun-protected body sites, risk factors other than direct solar irradiation are likely although these have not been identified. Indeed, a recent systematic review concluded that solar UVR is not an environmental risk factor for CM in people with skin of colour (51).

In contrast to the diseases outlined in the sections above, there is little evidence to indicate that outdoor workers, even with fair skins, have a higher risk than indoor workers or the general population of developing CM. A WHO Environmental Burden of Disease review included eight studies on the association between occupational sun exposure and CM, with only one of these reporting a positive relative risk for outdoor compared with indoor workers (21). Very recently a large cohort study based in Nordic countries (Denmark, Finland, Iceland, Norway and Sweden) assessed occupation and socioeconomic status with the number of CM cases during 1961–2005 (52). It was calculated that both men and women with outdoor work were at significantly lower risk of developing CM than those with indoor work. This was attributed, at least in part, to workers with very fair skin or with a known genetic risk of CM being less likely to be employed in outdoor occupations.

In brief, there is little or no evidence to link outdoor work with an increased risk of CM, irrespective of skin colour.

## Sun Protection Studies

The WHO has identified solar UVR as a hazard in the workplace (53) and recommends protecting workers from excess solar UVR exposure (54). Personal protective measures for people working outdoors are clothing, hats, sunscreens, eye protection and shade (54). The Ultraviolet Protection Factor (UPF) and Sun Protection Factor (SPF) were developed to assure users of the sun protection capabilities of clothing/hats and sunscreen, respectively. Typical methods of sun protection for outdoor workers include avoiding exposure to direct sunlight around midday, seeking shade, wearing clothing with high UPF, hats with broad brims as well as helmets with neck flaps, and eyewear with wrap-around design or side panels, applying broad-spectrum sunscreen with a SPF of at least 30 to all exposed body sites, and avoiding any unnecessary elective UVR exposure, such as from sunbed use (54).

A review in 2007 included 14 descriptive studies of sun exposure and sun protective behaviours in outdoor workers based mainly in USA and Canada (55). Preventive practices were variable but generally ineffective. Men were more likely than women to wear hats and protective clothing, but women were more likely to use sunscreen. Another review of 34 descriptive and 18 intervention studies of farmers, construction workers and aquatic personnel in USA, Canada and Australia revealed that occupational UVR exposure limits were frequently exceeded. Inadequate protective behaviour led to high sunburn rates (56).

With regard to South Africa, a survey of farm workers in Limpopo province found that 80% never wore sunglasses and 23% never wore a hat when working (57). When a hat was used, peak caps were preferred to broad-brimmed hats although the latter provided better sun protection. Farm workers in Upington in the Northern Cape province wore long-sleeved overalls as their uniform rather than for sun protection and complained that they felt extremely hot during warm weather (58). Forestry workers in the Western Cape protected their faces from the sun using a variety of substances including ochre, clay and ordinary hand lotion, along with broad-brimmed and hard hats (58).

It is important that sun protective measures used by workers should not impair or pose a hazard to their ability to conduct work tasks. Provision of sails and awnings for shade are important physical barriers against solar UVR exposure. For example, canopies and awnings may provide adequate sun protection. For workers trading in an informal street market in KwaZulu-Natal, portable shade in the form of gazebos and canopies was the most common form of sun protection (59).

**Figure 2:**
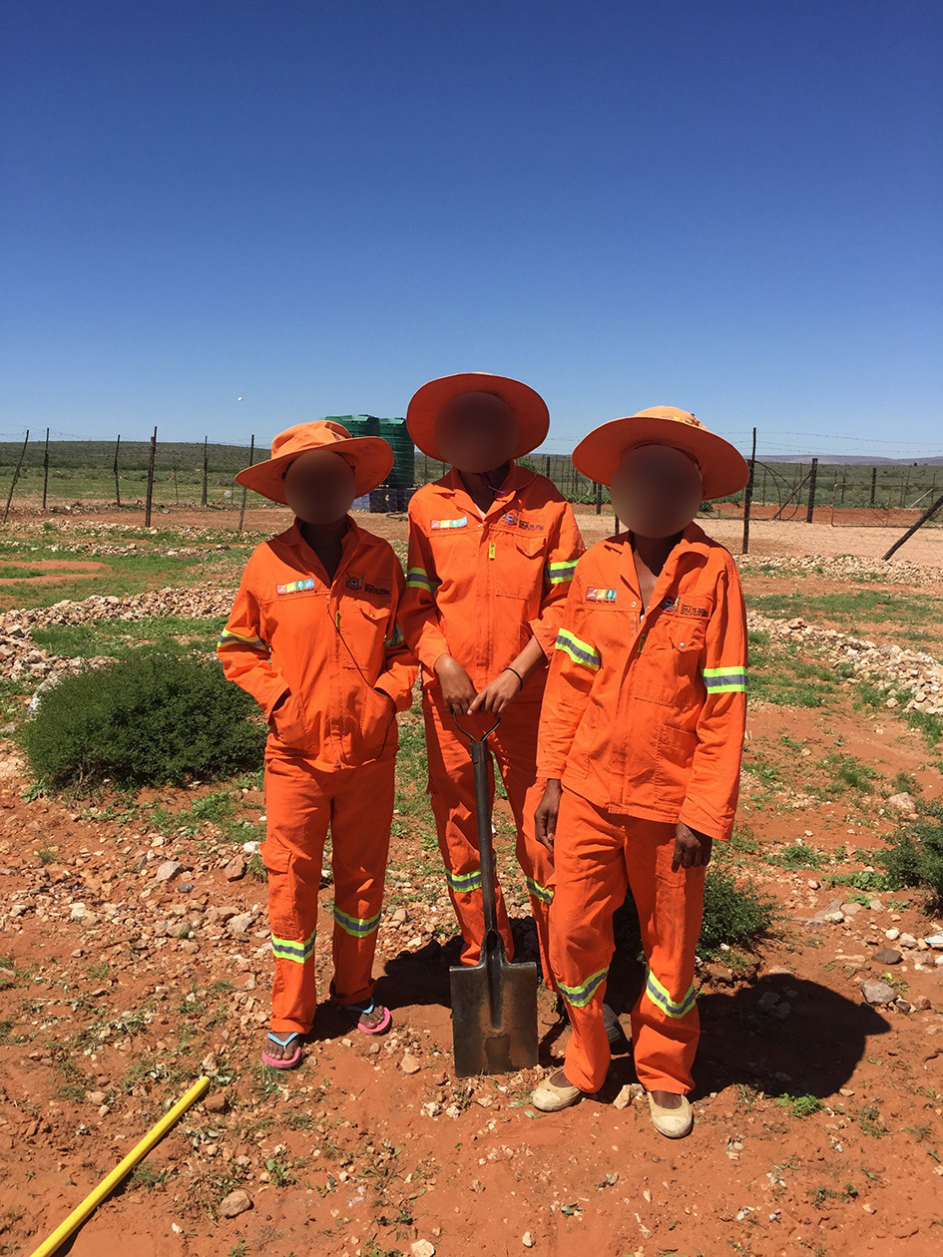
Outdoor workers wearing broad-brimmed hats and protective clothing in Groblershoop, the Northern Cape.

A critical determinant affecting the uptake of sun protection relates to personal knowledge, attitudes and behaviours. However, although outdoor workers in Germany knew about the risks of excess sun exposure, how to protect themselves, and what the UV Index means, such knowledge did not translate into sun protection uptake (60). Sunscreen application can be inappropriate and clothing uncomfortable or hinder the ability to conduct work tasks (61). Only one study in South Africa has considered knowledge and attitudes toward sun protection. Forestry workers in the Western Cape were aware of the risks of excess sun exposure but reported that they preferred not to use sunscreen because it was expensive and perceived to attract bees (58). Workers removed clothing when they felt hot, regardless of sun exposure, and chose not to use UVR protective goggles because they led to difficulties in seeing where to walk. Female workers wore broad-brimmed hats under their hard hats, while male workers had not been granted permission by the employer to do so (58). Female municipal gardeners working in Groblershoop in the Northern Cape wear broad-brimmed hats and protective clothing while working outdoors (Figure 2).

## Actions Needed to Ameliorate the Adverse Health Risks of Excess Solar UVR Exposure Policy/Guidelines

Several standards exist to limit artificial UVR exposure, such as the American Conference of Governmental Industrial Hygienists (ACGIH) limit, (62) the International Commission on Non-Ionizing Radiation Protection (ICNIRP) limit (63) and the Australian Radiation Protection Standard (64). However, no standards exist to limit solar UVR exposure of outdoor workers, probably due to the variability of solar UVR environments, behavioural effects and anatomical exposure geometry (65). The WHO in collaboration with ICNIRP and the International Labour Organization advocate protection of workers from solar UVR (54) and Australia has adopted similar occupational sun protection guidelines (65).

In Victoria, Australia, under state occupational health and safety legislation, it is considered a requirement that employers protect workers, including contracted and causal employees, from solar UVR exposure (66, 67). To ensure a safe UVR environment employers should have a solar UVR protection policy or guidelines in place stating control measures that are endorsed by senior management. They should provide information and training about solar UVR protection and provide solar UVR protection/control measures for employees. These include shade, modifying reflective surfaces, rescheduling outdoor programmes to avoid periods of high solar UVR and providing personal protective equipment, such as broad-brimmed hats, sunglasses, sunscreen and sun-protective clothing. Employees must co-operate with their employers' efforts to ensure protection from excessive sun exposure. Similarly, in Canada, the Occupational Health and Safety Regulations state that the employers should provide skin protection to their employees and make sun safety information prominent in the workplace (68).

Uptake of sun protection by outdoor workers, when sun protection policy/guidelines do not exist, is generally poor (69, 70). In the mining sector, risk assessments (71, 72) are conducted and some include consideration of exposure to solar UVR and provisions of recommendations for exposure management and protective measures.

However, in general, in South Africa there are no national policies or guidelines for employers on how to protect outdoor workers from excess solar UVR exposure nor the most effective methods for the employees to protect themselves. This gap needs to be addressed by first amending the Occupational Health and Safety Amendment Act (No. 181 of 1993) (73) to include solar UVR exposure as an occupational risk. The National Institute for Occupational Health together with the Cancer Association of South Africa (CANSA) would be appropriately positioned to draft workplace sun safety guidelines that present the case for sun protection at work, important facts about solar UVR, mechanisms to protect workers from adverse sun exposure impacts, and health surveillance in the workplace (74). Guidelines should follow those recommended by the WHO (53) and should include at least the following sections:

- A description of what solar UVR is and why it is a hazard in the workplace;
- The health risks associated with exposure to solar UVR in the workplace, including effects on the skin and the eyes;
- How to manage the risks associated with excess solar UVR exposure in the workplace using several measures including:
  ° Engineering controls: e.g., shade cover.
  ° Administrative controls: e.g., rescheduling outdoor work programmes to avoid peak solar UVR hours.
  ° Personal protective equipment: e.g., hard hats with neck flaps, sunglasses.
  ° Training: e.g., on the risks of excess exposure to solar UVR and what is expected of employers and employees while at the workplace to minimize the risks.
- What to do if workers have been overexposed, i.e., to seek medical attention.

These generic guidelines should be tailored for different sectors and types of outdoor work, as well as for geographic location which influences solar UVR intensity (2) and workplace culture (70) to ensure commitment and uptake by outdoor workers. The South African Institute of Occupational Safety and Health (Saiosh) is the membership body which could assist with knowledge dissemination of the proposed guidelines as well as training of occupational hygienists on sun protection in the workplace.

## Employee/Employer Education and Training

The UV Index is a tool that can be used by outdoor workers to understand when solar UVR levels are deemed to be risky; sun protection is required when the UV Index is 3 or greater (2). A UV Index of 3–5 (moderate) calls for taking precautions when outdoors such as covering up, using sunscreen and staying in the shade the during midday hours. When the UV Index is high (UV Index 6–7) workers are advised to adjust their work schedules to avoid exposure between 11 h and 16 h and use sun protection, i.e., clothing, hat, shade, sunglasses, and sunscreen. Very high (UV Index 8–9) and extreme (UV Index 11+) values call for workers who must work outside to take all precautions since unprotected skin and eyes can burn quickly.

Several countries have developed training materials for occupational sun protection including the Health and Safety Agency in the United Kingdom, (75) the Australian Radiation Protection and Nuclear Safety Agency, (76) and the United States Department of Labour (77). Safety, Health, Environment and Quality (SHEQ) training should include information about relevant health risks and the need to protect the eyes and skin. When policy/guidelines, educational interventions and sun protection are implemented in the workplace, there is strong evidence that skin cancer and other solar UVR exposure-related health risks in outdoor workers can be reduced (78).

In summary, the South African Occupational Health and Safety Amendment Act (No. 181 of 1993) (73) provides for workers' rights to a safe and healthy occupational environment. However, there is no specific legislation regarding solar UVR exposure for outdoor workers **(**such as those engaged in agriculture, forestry or construction) in South Africa. Moreover, little attention is paid to occupational health in the country's climate change and health adaptation plan (79). South Africa needs to amend its occupational health and safety legislation by acknowledging solar UVR exposure as an occupational risk. Is also needs to consider developing and implementing sun safety guidelines and training modules that inform workers and employers about the health risks associated with excessive sun exposure in the workplace and appropriate sun protection measures.

## Conclusion and Recommendations

Due to high ambient solar UVR levels throughout much of the year in South Africa, there is the potential for an increased risk of several eye and skin diseases in outdoor workers. Although few studies have examined this possibility in South Africa, strong evidence from round the world has been obtained. Detailed results are discussed which demonstrate the association between solar irradiation and an increased incidence of acute sunburn of the eyes and skin, and of the chronic conditions, pterygium, cataract and skin cancer in those who work outdoors compared with indoor workers or the general population. Future research in South Africa should determine solar UVR-associated health impacts among workers in different sectors, especially for the skin and eyes.

Sun protection is an effective way to reduce solar UVR exposure for those working outside. Several countries have developed policies and guidelines to promote sun safety in the workplace. These include training, personal protective equipment and managerial support. In South Africa, legislation is needed to recognise solar UVR exposure as an occupational health hazard, with sun safety guidelines and training provided for both employers and employees.

## Lessons Learned

- South Africa experiences high solar ultraviolet radiation levels that pose health risks.
- Outdoor workers are at risk of high personal sun exposure that may affect their eyes and skin.
- South African policy and/or legislation needs to recognise sun exposure risks for workers.
- Employers and employees should apply appropriate sun protection measures.

## Author Contributions

All authors listed have made a substantial, direct and intellectual contribution to the work, and approved it for publication.

## Conflict of Interest

The authors declare that the research was conducted in the absence of any commercial or financial relationships that could be construed as a potential conflict of interest.
